# Intervention effects and long-term changes in physical activity and cardiometabolic outcomes among children at risk of noncommunicable diseases in South Africa: a cluster-randomized controlled trial and follow-up analysis

**DOI:** 10.3389/fpubh.2023.1199381

**Published:** 2023-05-26

**Authors:** Patricia Arnaiz, Harald Seelig, Markus Gerber, Larissa Adams, Jan Degen, Danielle Dolley, Nandi Joubert, Madeleine Nienaber, Siphesihle Nqweniso, Peter Steinmann, Jürg Utzinger, Rosa du Randt, Cheryl Walter, Uwe Pühse, Ivan Müller

**Affiliations:** ^1^Department of Sport, Exercise and Health, University of Basel, Basel, Switzerland; ^2^Department of Human Movement Science, Nelson Mandela University, Gqeberha, South Africa; ^3^Swiss Tropical and Public Health Institute, Allschwil, Switzerland; ^4^University of Basel, Basel, Switzerland

**Keywords:** physical activity, health promotion, school-based, noncommunicable diseases, effectiveness, sustainability, South Africa

## Abstract

**Introduction:**

Risk factors for noncommunicable diseases such as insufficient physical activity (PA), overweight or hypertension are becoming increasingly predominant among children globally. While school-based interventions are promising preventive strategies, evidence of their long-term effectiveness, especially among vulnerable populations, is scarce. We aim to assess the short-term effects of the physical and health *KaziKidz* intervention on cardiometabolic risk factors and the long-term, pre-and post-COVID-19 pandemic changes thereof in high-risk children from marginalized communities.

**Methods:**

The intervention was tested in a cluster-randomized controlled trial between January and October 2019 in eight primary schools near Gqeberha, South Africa. Children with overweight, elevated blood pressure, pre-diabetes, and/or borderline dyslipidemia were identified and re-assessed 2 years post-intervention. Study outcomes included accelerometry-measured PA (MVPA), body mass index (BMI), mean arterial pressure (MAP), glucose (HbA1c), and lipid levels (TC to HDL ratio). We conducted mixed regression analyses to assess intervention effects by cardiometabolic risk profile, and Wilcoxon signed-rank tests to evaluate longitudinal changes in the high-risk subpopulation.

**Results:**

We found a significant intervention effect on MVPA during school hours for physically inactive children, and among active as well as inactive girls. In contrast, the intervention lowered HbA1c and TC to HDL ratio only in children with glucose or lipid values within the norm, respectively. At follow-up, the intervention effects were not maintained in at-risk children, who showed a decline in MVPA, and an increase in BMI-for-age, MAP, HbA1c and TC to HDL ratio.

**Conclusion:**

We conclude that schools are key settings in which to promote PA and improve health; however, structural changes are necessary to ensure that effective interventions reach marginalized school populations and achieve sustainable impact.

## 1. Introduction

Unhealthy behaviors, such as physical inactivity, and subsequent cardiometabolic changes including increased weight, raised blood pressure (BP), high plasma glucose, or an abnormal lipid profile are well-established risk factors for noncommunicable diseases (NCDs) diseases (1). While NCDs appear typically in adulthood, evidence shows that health during the first periods of life will have an impact on adult life (2). Yet, modifiable risk factors are getting more predominant among children. For instance, it is estimated that 81% of children worldwide do not meet the recommended 60 min of moderate to vigorous intensity physical activity (MVPA) per day (3). Meanwhile, childhood obesity and hypertension have increased dramatically in the last decades (4, 5). Hence, reducing risk factors early in life to prevent the continuous rise of NCDs is urgently needed.

One strategy to halt the incidence of NCDs is promoting healthy, active lifestyles from a young age (6). Engagement in regular physical activity (PA) has been described to support physical and mental health in school-aged children and avert weight-related cardiometabolic diseases (7–10). At the same time, education to support behavioral change around PA has been recognized as a cost-effective strategy to reduce NCDs (11). Thus, several PA interventions have focused on the school setting for this purpose. Concretely, comprehensive school PA programs (CSPAs) have been identified as promising strategies to increase daily movement while reaching vast numbers of children (12).

However, conflicting literature exists about the effectiveness of school-embedded programs in increasing PA and improving health among children. Systematic reviews have demonstrated either no improvement in PA and cardiovascular risk (13, 14), or positive though modest effects (15, 16) both in the short- and long-term. Although initial beneficial effects have been described to attenuate over time, more complex interventions that showed no immediate impact reported improvements long after the intervention’s end (17). Thus, follow-up measurements are important to evaluate the efficacy of interventions and maintenance of results.

Furthermore, the majority of evidence originates from high-income countries. In South Africa, varying results have been described for school-based interventions on health behaviors and risk among underprivileged adolescents (18, 19). A recent systematic review corroborated the inconsistency in interventions effectiveness in Africa (20). On the other hand, studies have shown that tailoring programs to the needs of vulnerable groups rather than prioritizing a higher population reach, increases intervention feasibility (21). The scarcity of evidence and discrepant results highlight the need for further research on the long-term effectiveness of lifestyle interventions in low- to middle-income countries, especially among vulnerable groups.

Against this background, we developed the *KaziKidz* health promotion program incorporating CSPAs components and tailoring it to low-resourced schools.^1^ The main purpose of this study is to examine short- and long-term changes on the cardiometabolic risk profile of children at risk of NCDs as a result of program participation. Specifically, we will first compare children presenting risk factors for NCDs with healthy counterparts, and assess the intervention effect on movement and cardiometabolic parameters. We will then examine the development of these risk factors in at-risk children two years’ post-intervention. Based on prior knowledge, we hypothesize that the intervention will have a positive effect on improving learners’ health outcomes, but we expect the effect to decline over time.

## 2. Materials and methods

### 2.1. Study design

A cluster-randomized controlled trial (RCT) was implemented in low-income primary schools from marginalized neighborhoods around Gqeberha, in the Eastern Cape of South Africa (22). The study included baseline assessments in January 2019, a 20 week intervention period, and post-intervention measurements in October 2019. Eight schools met the inclusion criteria of geographical location and representativeness, language, and school commitment. The allocation to a study condition was done at the school level, in a sequential manner, and using opaque, sealed envelopes. Four schools were randomly allotted to the intervention arm, and four to the control arm.

Building on the RCT, a cohort of at-risk children was identified and followed up two years’ post-intervention with a final assessment in October 2021.

### 2.2. Intervention

The *KaziKidz* intervention is part of the *KaziBantu* project that aims to enhance health and physical literacy in low-resourced primary schools. It utilizes the *KaziKidz* toolkit, which consists of ready-to-use lessons for teachers covering (1) physical education (PE), (2) moving-to-music dance classes, and (3) health, hygiene and nutrition education. PE and moving-to-music each comprise 32 lessons of 40 min, with one weekly lesson of each subject. Health and hygiene, and nutrition each include three 40 min lessons conducted throughout the school year. All lessons are adapted for grades one to seven.

The *KaziKidz* teaching material was delivered to all classes grades four to six from March to July 2019 by life-skills orientation teachers. In addition, the four intervention schools received basic sports equipment and painted playground games. To test different delivery strategies, two of these schools were additionally offered two 90 min workshops that informed teachers on the practical implementation of the toolkit. One of them further received support from a trained sports graduate from the Department of Human Movement Science at the Nelson Mandela University, who assisted teachers with the intervention delivery.

Further details on the intervention can be found in the study protocol (22).

### 2.3. Participants

In the original RCT, data was collected from one class of each grade (grades four to six) in all participating schools, with most children being 8–13 years old. For a child to participate in the RCT, the following inclusion criteria had to be met: (1) having written consent from a guardian and oral assent from child, (2) not been enrolled in other clinical trials, and (3) have been cleared to participate by a qualified healthcare professional.

**Figure 1:**
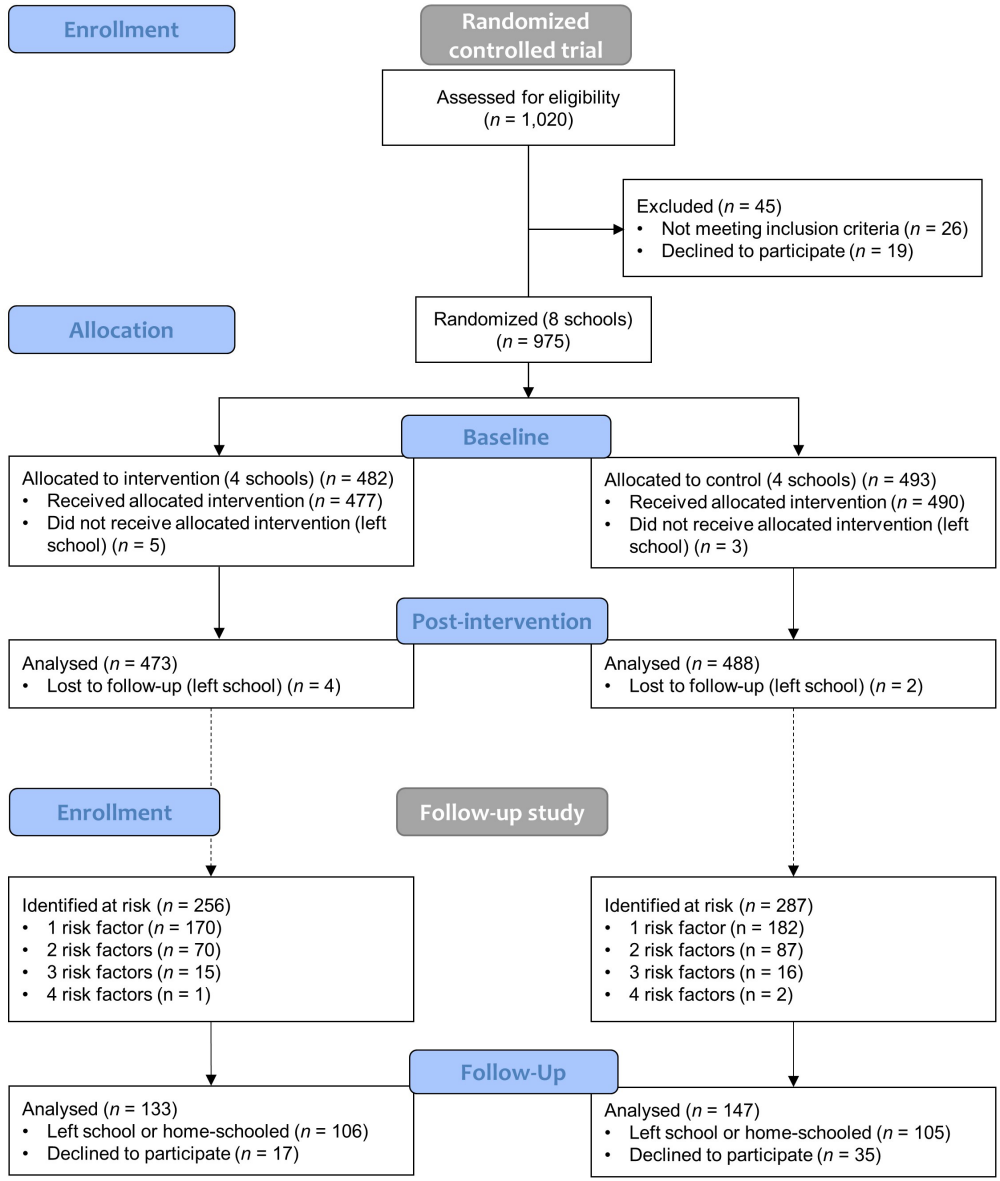
Participant flow diagram.

As reported in the first section of the flow diagram (Figure 1), 1,020 learners were screened and 975 randomized at school level after having met the inclusion criteria. Excluding baseline and post-intervention drop-outs, data from 961 learners (473 intervention versus 488 control arm) were included in the short-term analysis.

For the follow-up study, a sub-cohort of children was selected and included in the study if they (1) had participated in the RCT baseline and post-intervention assessments; (2) based on the baseline records presented at least one of the following cardiometabolic diseases: (i) overweight or obesity, (ii) elevated BP or hypertension, (iii) pre-diabetes or diabetes, (iv) borderline or dyslipidemia, based on age-standardized guidelines; and (3) had given consent. The second section of Figure 1 shows that from an eligible sample of 543 children identified at risk, 280 were enrolled in the study and included in the final analysis (133 intervention versus 147 control arm). Further details on the study population and setting can be found in the study protocols (22, 23).

### 2.4. Data collection

#### 2.4.1. Physical activity

PA was assessed via accelerometry. The triaxial actigraph wGT3X-BT (ActiGraph LLC, Pensacola, United States) was set up at a 30 Hz sampling rate and worn around the hip for seven consecutive days. Data processing was done in the ActiLife software (version 6.13.4; ActiGraph LLC, Pensacola, United States) using a 10 s epoch length and the Troiano et al. algorithm to remove non-wear-time (24). PA measurements were included for further analyses if they consisted of at least three valid schooldays and one valid weekend day. A valid day was defined by a minimum of three hours’ wear-time between 08:00 and 15:00 (school days), or eight hours between 06:00 and 24:00 (weekend day). MVPA was calculated as minutes per day spent in moderate and vigorous intensity activity levels, as defined by Evenson et al. cut-off points. Non-compliance with the 60 min of MVPA per day recommended for children by the World Health Organization (WHO) was considered physical inactivity.

#### 2.4.2. Anthropometric and clinical outcomes

We used a digital weighing scale (MC-580; Tanita, Tokyo, Japan) to measure body weight and a stadiometer for body height. Body mass index represents weight (kg) divided by squared height (m^2^). Sex-adjusted BMI-for-age *z* scores (zBMI) were determined based on the WHO children growth standards (25). Overweight was defined as zBMI values over 1 standard deviation.

Resting BP was appraised three times using the Omron automated oscillometric device (Omron^®^ M6 AC; Hoofddorp, Netherlands). The mean of the last two readings was used to compute systolic (SBP) and diastolic (DBP) BP. Mean arterial blood pressure (MAP) was calculated as: 1/3(SBP-DBP) + DBP (26). A SBP and/or DBP over the 90th percentile or 120/80 mmHg were characterized as elevated BP (27).

Minimally invasive blood sampling was done by pricking the child’s fingertip with a safety lancet. The Alere Afinion AS 100 Analyzer device (Abbott Laboratories, Illinois, United States) was used to determine glycated haemoglobin (HbA1c) and total cholesterol (TC) to high-density lipoprotein (HDL) ratio levels. HbA1c values higher than 39 mmol/mol and a TC higher than 4.40 mmol/L were indicative of pre-diabetes (28) and borderline dyslipidemia (29), respectively.

#### 2.4.3. Socioeconomic status

We employed a nine-item questionnaire covering housing characteristics (type of house, number of bedrooms, own toilet, type of toilet, access to water, access to electricity) and household possessions (washing machine, refrigerator, car) to evaluate the socioeconomic status (SES) of children according to previous research (30). A SES index was calculated by dichotomizing the nine items (0 = not available, low quality; 1 = available, high quality) and adding them up, whereby 0 represents the lowest SES possible and 9 the highest.

### 2.5. Statistical analyses

Data was double-entered and validated using EpiData (version 3.1), and EvaSys (version 7.1) was used to appraise questionnaire data. Continuous outcomes were described with medians and 95% confidence intervals (CI), while categorical variables were presented as frequencies and 95% CI. Since missing data differed by outcome, we reported sample size for each analysis separately. We applied a series of separate linear mixed models to evaluate the short-term intervention effect on PA and health outcomes by risk group using classes as random effects. Model for zBMI-for-age was controlled for baseline levels and SES. Models for MAP, HbA1c and TC to HDL ratio were controlled for baseline levels, age, sex, and SES. Total and school MVPA were controlled for baseline levels, age, sex, SES, and wear-time. Post-hoc analyses included studying the effect of the intervention on PA levels by sex. To assess the long-term development of cardiovascular risk factors in the subsample of at-risk children, we performed pair-wise Wilcoxon signed-rank tests for within-subject variations between baseline, post-intervention and follow-up. Size effect r was calculated as the absolute *z* score divided by the square root of the sample size, and interpreted according to Cohen (31). We then conducted the mixed linear regression models described above to examine the association between intervention arm and long-term outcomes. We used the bias-corrected and accelerated bootstrap interval with 1,000 replicates to construct estimates and 95% CI, and we set a significance level at *p* < 0.05 for all analyses. Statistical analyses were done in IBM SPSS Statistics (version 28) for Windows.

## 3. Results

Table 1 shows baseline characteristics of all participants by intervention group. Median age was 10.8 years old (95% CI: 10.71 to 10.92) and the percentage of girls and boys was distributed almost equally between both the intervention and the control group (girls: 49.0% versus 48.8%; boys: 51.0% versus 51.2%, respectively). Median SES was 6.0 (95% CI: 6.00 to 6.00) for both groups. The control and the intervention groups differed in MAP (Md: 80.67 versus 79.00, respectively) and TC to HDL ratio (Md: 3.00 versus 2.80, respectively). The prevalence of risk factors for all participants was 22.4% (95% CI: 19.7 to 25.2%) for overweight or obesity, 36.7% (95% CI: 33.6 to 39.8%) for elevated BP or hypertension, 13.6% (95% CI: 11.1 to 16.2%) for pre- or diabetes, 16.0% (95% CI: 13.2 to 18.6%) for pre- or dyslipidemia, and 35.8% (95% CI: 32.7 to 39.3) for physical inactivity.

**Table 1:** Demographic and clinical characteristics of participants at baseline, in January 2019 (*N* = 961).

Numeric variable	Control			Intervention		
	*N*	Median	95% CI	*N*	Median	95% CI
Age (years)	488	10.88	10.72–11.02	473	10.73	10.61–10.89
Total MVPA^a^ (min/day)	466	67.82	64.61–71.42	449	71.93	68.17–74.24
In-school MVPA (min/day)	466	16.26	15.33–17.20	449	16.60	15.93–17.50
BMI^b^-for-age (*z* scores)	443	0.15	0.00–0.28	469	−0.12	−0.23–0.01
MAP^c^ (mmHg)	478	**80.67**	**79.67–81.50**	460	**79.00**	**78.17–79.67**
HbA1c^d^ (mmol/mol)	364	36.00	35.00–36.00	395	36.00	35.00–36.00
TC:HDL^e^	357	**3.00**	**2.90–3.10**	394	**2.80**	**2.70–2.90**
SES^f^ (0–9 scale)	454	6.00	6.00–7.00	435	6.00	6.00–6.00

Categorical variable	Control			Intervention		
	*N*	%	95% CI	*N*	%	95% CI
Sex						
Girls	238	48.8	44.5–53.3	232	49.0	44.4–53.7
Boys	250	51.2	46.7–55.5	241	51.0	46.3–55.6
Physical inactivity^g^	176	37.8	33.5–42.3	152	33.9	29.8–38.3
Overweight^h^	113	25.5	21.2–29.8	91	19.4	15.8–22.8
Elevated blood pressure^i^	183	38.3	33.9–42.5	161	35.0	30.7–39.6
Prediabetes^j^	50	13.7	10.4–17.3	53	13.4	10.1–16.7
Predyslipidemia^k^	66	18.5	14.6–22.7	54	13.7	10.2–17.3

a Moderate to vigorous intensity physical activity.

b Body mass index.

c Mean arterial pressure.

d Glycated haemoglobin.

e Total cholesterol to high-density lipoprotein ratio.

f Socioeconomic status.

g MVPA below 60 min a day.

h zBMI-for-age over 1 standard deviation.

i Systolic and/or diastolic blood pressure over the 90th percentile or 120/80 mmHg.

j HbA1c over 39 mmol/mol.

k TC over 4.40 mmol/L.

Bold values indicate statistical significance.

Results of mixed regression analyses are presented in Table 2. The intervention showed a statistically significant effect in increasing MVPA during school among physically inactive children (*B* = 1.71, 95% CI: 0.14 to 3.35, *p* = 0.008) but not in already active children (*B* = 0.19, 95% CI: −1.58 to 1.08, *p* = 0.762). No significant intervention effect was observed for total MVPA in neither group. The intervention significantly lowered MAP both in children at risk of hypertension (*B* = −2.16, 95% CI: −4.20 to −0.06, *p* = 0.008) and in those not at risk (*B* = −1.77, 95% CI: −3.14 to −0.70, *p* = 0.004). For both HbA1c and TC to HDL ratio a significant positive intervention effect was observed in children not at risk of diabetes (HbA1c: *B* = −0.26, 95% CI: −0.52 to −0.01, *p* = 0.037) or dyslipidemia (TC to HDL ratio: *B* = −0.11, 95% CI: 0.18 to −0.05, *p* = 0.002). Meanwhile, children with higher HbA1c baseline levels did not experience a significant intervention effect (*B* = −0.38, 95% CI: −1.32 to 0.29, *p* = 0.458), while the intervention significantly increased TC to HDL ratio levels compared with those at baseline (*B* = 0.12, 95% CI: −0.002 to 0.23, *p* = 0.036).

**Table 2:** Post- intervention effects on cardiometabolic risk factors in not at-risk versus at-risk children.

Risk factor	Not at-risk				At-risk			
	*N*	Beta	95% CI	*p*-value	*N*	Beta	95% CI	*p*-value
Total MVPA^a^ (min/day)	425	−1.64	−5.94–2.66	0.314	254	0.70	−3.72–5.85	0.643
In-school MVPA (min/day)	425	−0.19	−1.58–1.08	0.762	253	**1.71**	**0.14–3.35**	**0.008**
BMI-for-age^b^ (*z* scores)	646	−0.02	−0.07–0.03	0.336	186	−0.001	−0.09–0.11	0.981
MAP^c^ (mmHg)	538	**−1.77**	**−3.14–** **−0.70**	**0.004**	317	**−2.16**	**−4.20–** **−0.06**	**0.008**
HbA1c^d^ (mmol/mol)	600	**−0.26**	**−0.52–** **−0.01**	**0.037**	97	−0.38	−1.32–0.29	0.458
TC:HDL^e^	576	**−0.11**	**−0.18–** **−0.05**	**0.002**	111	**0.12**	**−0.002–** **0.23**	**0.036**

At-risk is defined for each outcome separately as: physical inactivity for total and in-school MVPA, overweight or obesity for BMI-for-age, elevated blood pressure or hypertension for MAP, pre- or diabetes for HbA1c, pre- or dyslipidemia for TC:HDL. Mixed linear regression were applied. All outcomes have been controlled for baseline outcome, age, sex and SES using class as random effects; BMI-for-age has not been controlled for age and sex; total and school MVPA have been further controlled for wear-time. Bias-corrected and accelerated (BCa) and 1,000 replicates bootstrap adjusted results.

a Moderate to vigorous intensity physical activity.

b Body mass index.

c Mean arterial pressure.

d Glycated haemoglobin.

e Total cholesterol to high-density lipoprotein ratio.

Bold values indicate statistical significance.

The regression analysis showed a significant association of sex with total and in-school MVPA. Post-hoc analyses revealed that the intervention was effective in increasing in-school MVPA time among girls both not compliant (*B* = 2.03, 95% CI: 0.58 to 3.42, *p* < 0.001) and compliant (*B* = 1.80, 95% CI: −0.22 to 3.82, *p* = 0.035) with PA recommendations, but no significant association was observed for boys in either group (*B* = 0.68, 95% CI: −5.39 to 7.82, *p* = 0.694; *B* = −1.39, 95% CI: −3.29 to 0.50, *p* = 0.109, respectively), nor in total MVPA for any sub-group (Supplementary Table S1).

We report Wilcoxon signed-rank findings for within-subject variations over time in at-risk children in Table 3. In general, the test revealed a statistically significant change in all outcomes during the intervention period, and no significant change between post-intervention and follow-up. In physically inactive children, both total and in-school MVPA increased significantly (*z* = −4.24, *p* < 0.001; *z* = −3.74, *p* < 0.001, respectively) from baseline to post-intervention. From post-intervention to follow-up, the median score of total MVPA declined from 53.29 to 41.90, although this decrease was not statistically significant (*z* = −1.95, *p* = 0.05) and of small effect size (*r* = 0.26). For in-school MVPA, a non-significant increase of small effect was observed at follow-up (*z* = 1.45, *p* = 0.15, *r* = 0.17). MAP was the only outcome that showed significant changes in both periods. While a significant decrease of large size effect was observed during the intervention (*z* = −6.48, *p* < 0.001, *r* = 0.53), MAP levels increased between post-intervention and follow-up (*z* = −5.18, *p* < 0.001) with a medium size effect (*r* = 0.42). Similarly, a statistically significant reduction of large size effect was observed from baseline to post-intervention in both HbA1c (*z* = −5.78, *p* < 0.001, *r* = 0.88) and TC to HDL ratio (*z* = −4.71, *p* < 0.001, *r* = 0.62), followed by a rise of the median values at follow-up that was non-significant and of small effect size (HbA1c: *z* = −1.63, *p* = 0.104, *r* = 0.25; TC to HDL ratio: *z* = −1.61, *p* = 0.11, *r* = 0.21). Median zBMI-for-age raised over time across all time points (Md: baseline: 1.76, post-intervention: 1.77, follow-up: 1.82).

**Table 3:** Longitudinal changes in cardiometabolic risk factors in children at risk of noncommunicable diseases from baseline to follow-up.

Risk factor	*N*	Baseline	Post-intervention	Follow-up	Baseline—post-intervention			Post-intervention—follow-up		
		Median (95% CI)	Median (95% CI)	Median (95% CI)	*z*	*p*-value	*r*	*z*	*p*-value	*r*
Total MVPA^a^ (min/day)	58	46.65 (45.71–48.60)	53.29 (49.40–57.26)	41.90 (38.40–53.31)	**−4.24**	**<0.001**	**0.56**	−1.95	0.052	0.26
In-school MVPA (min/day)	74	12.65 (11.50–15.03)	15.88 (14.13–17.93)	17.73 (15.53–19.47)	**−3.74**	**<0.001**	**0.43**	−1.45	0.147	0.17
BMI-for-age^b^ (z scores)	101	1.76 (1.41–1.99)	1.77 (1.57–2.10)	1.82 (1.60–2.10)	**−2.07**	**0.039**	**0.21**	−0.34	0.737	0.03
MAP^c^ (mmHg)	152	87.75 (86.08–89.50)	81.92 (80.58–83.83)	85.75 (84.25–87.50)	**−6.48**	**<0.001**	**0.53**	**−5.18**	**<0.001**	**0.42**
HbA1c^d^ (mmol/mol)	43	39.00 (39.00–40.00)	37.00 (36.00–37.00)	38.00 (37.00–38.00)	**−5.78**	**<0.001**	**0.88**	−1.63	0.104	0.25
TC:HDL^e^	57	3.30 (3.00–3.50)	2.90 (2.70–3.30)	3.00 (2.80–3.20)	**−4.71**	**<0.001**	**0.62**	−1.61	0.11	0.21

At-risk is defined for each outcome separately as: below 60 min of MVPA a day for total and in-school MVPA, overweight or obesity for BMI-for-age, elevated blood pressure or hypertension for MAP, pre- or diabetes for HbA1c, pre- or dyslipidemia for TC:HDL. Wilcoxon signed-rank test*. r* = *z*/√*N*. Interpretation of size effect according to Cohen (1988): Small: *r* from 0.01 to 0.029. Medium: *r* from 0.30 to 0.49. Large: *r* ≥ 0.5. Bias-corrected and accelerated (BCa) and 1,000 replicates bootstrap adjusted results.

a Moderate to vigorous intensity physical activity.

b Body mass index.

c Mean arterial pressure.

d Glycated haemoglobin.

e Total cholesterol to high-density lipoprotein ratio.

Bold values indicate statistical significance.

**Figure 2:**
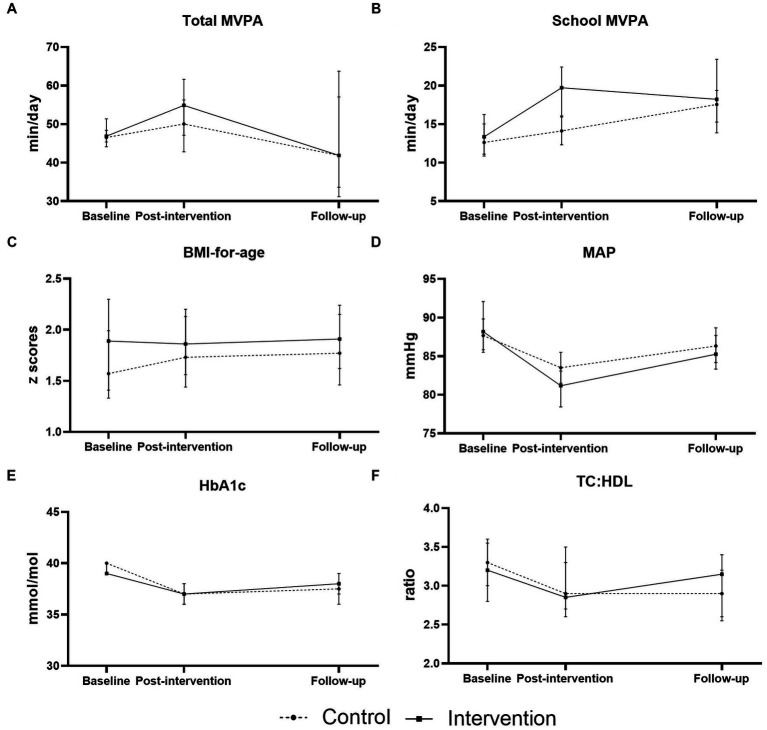
Longitudinal changes in cardiometabolic risk factors in children at risk of noncommunicable diseases. Changes from baseline to post-intervention and from post-intervention to follow-up are represented by intervention arm for **(A)** total moderate to vigorous intensity physical activity (total MVPA), **(B)** in-school moderate to vigorous intensity physical activity (school MVPA), **(C)** sex- and age-adjusted body-mass-index (BMI-for-age), **(D)** mean arterial pressure (MAP), **(E)** glycated haemoglobin (HbA1c), and **(F)** total cholesterol to high-density lipoprotein ratio (TC:HDL).

Further, we wanted to examine whether an intervention effect could be observed in the long-term. Figure 2 depicts the development of cardiovascular risk factors in at-risk children from baseline to follow-up by intervention arm. For at-risk children who participated in the intervention, PA levels decreased between post-intervention and follow-up, and all other risk factors increased. Children at risk who were in the control group followed a similar development, except for school MVPA, whose levels rose during this period. We confirmed these observations via mixed regression analyses (Supplementary Table S2). No significant intervention effect was found between post-intervention and follow-up.

## 4. Discussion

The main findings of this study are that the *KaziKidz* school-based intervention had beneficial short-term effects that differed depending on the children’s cardiometabolic risk profile, and that the observed effects lessened over time in vulnerable children. In the short-term, the intervention was effective in increasing during school MVPA for physically inactive children and among girls regardless of initial PA levels. At the same time, the intervention reduced MAP for all children, while only healthy children’s HbA1c and TC to HDL ratio levels decreased significantly. At the 2 year follow-up, the intervention effects were not maintained in children at risk of NCDs, who showed lower total time spent in MVPA and elevated zBMI-for-age, MAP, HbA1c and TC to HDL ratio levels.

Our first aim was to study the immediate effect of a health-enhancing physical education intervention on cardiometabolic indicators in children at risk of NCDs compared to their healthy peers.

Although mixed results have been reported for school-based programs targeting PA, our findings are in line with those of a recent umbrella review (32). In this overview, Mannocci and colleagues found small increases in PA among children. However, the significant improvement in-school MVPA was not observed in total MVPA. Disagreement between in-school PA participation and total movement throughout the day has already been described. Favorable effects on PA levels during PE classes and school hours were not mirrored by a positive impact during leisure time (16), or were attenuated by inactivity after school (14). So far, the focus around the health benefits of PA has been on total MVPA across the day. We argue that neglecting interventions that prove effective in increasing MVPA during the school time would be a missed opportunity to reach certain subgroups with less inherent motivation or opportunities to move outside of school hours.

In fact, the positive results found on school MVPA were on children with low initial levels of PA, as well as in girls independent of their activity status. These findings differ from that of a pooled analysis that detected a positive effect on PA for already active children, but not for inactive children or girls (33). Similarly, Love et al. did not find evidence of variable efficacy by sex (14). One possible explanation is that our intervention was better suited to meet the specific needs of inactive children and girls compared to the studies included in these analyses. Concretely, the *KaziKidz* toolkit aims to integrate movement into the learners daily lives through fun and games, rather than with competitive team games. The latest has been identified as a barrier of girls’ participation in physical activities (34). Moreover, increasing movement levels in these groups could help develop their motoric skills and, ideally, create long-lasting positive attitudes towards PA. As high-intensity PA has been repeatedly associated with better cardiovascular outcomes (35), even small increases in PA levels accumulated over time could have meaningful clinical implications in at-risk populations.

Conflicting results exist about the potential of interventions to modify cardiometabolic risk markers. In our study, the intervention did not affect BMI levels in neither normal weight or overweight learners. However, it lowered MAP for children with and without pre-existing elevated BP. Furthermore, a significant improvement in glucose and lipid levels was only observed among children with no glycemic or lipidemic disorders, respectively. Poor cardiovascular health in children is oftentimes associated with unhealthy habits. Changing at-risk behaviors through lifestyle interventions has proven challenging. For example, limited results in BMI reduction among severely obese children and sustainability issues have been described (36). It is possible that longer intervention duration or higher intervention intensity might be needed to see an improvement, or that our intervention did not meet the specific needs of children with existing health disorders. Considering that high-risk subgroups are more susceptible to adverse health outcomes across the life-course, more research is imperative to identify effective and sustainable interventions in these groups.

The second aim of the study was to assess the long-term development of risk factors in especially vulnerable children. In general, the cardiometabolic risk profile of at-risk children worsened over time. However, results must be interpreted in the context of the COVID-19 pandemic, as South Africa employed one of the strictest lockdown measures globally. The 2 year follow-up assessments took place after children re-entered schools and while operating at 50% capacity.

A decline in total MVPA of over 11 min/day, although not statistically significant, was observed from October 2019 to October 2021. This reduction is higher than the 8 min/day reported for a population of school-aged English children between Summer 2018 and Autumn 2021 in the United Kingdom, which would be broadly equivalent to the expected natural decline observed with age (37). Consequently, we saw an increase of almost 10% in the prevalence of physical inactivity compared to baseline. Interestingly, the median school MVPA increased. We speculate that families maintained limited physical and social contact after COVID-19-related restrictions were lifted, limiting children’s playtime outdoors. Meanwhile, schools would present as an alternative space for free and safe movement, and play. Our results point towards a longstanding adverse effect of the COVID-19 pandemic on PA levels, and potentially other cardiometabolic risk factors.

Indeed, a deterioration of children’s health during the COVID-19 pandemic has been reported by other authors, although evidence is limited and heterogeneous. In our study we found a significant increase in MAP at follow-up, and small, non-significant changes in HbA1c and TC to HDL ratio. Our observations are in line with one study that found significant changes in HbA1c and lipid levels among dyslipidemic children (38). Another study in children with type 1 diabetes found a generalized decline in cardiometabolic risk factors including lipid parameters (39). Furthermore, an increase in pediatric type 2 diabetes during the COVID-19 pandemic has been reported (40). The higher consumption of salty and sweet foods and beverages noted during lockdown periods (41), may be one cause of health decline. Moreover, because PA is inversely associated with cardiometabolic risk factors (42), it is reasonable to think that the decline in total MVPA might partly explain the raise in BP, glucose and lipid levels observed in our study population. Notably, we did not see a significant increase in zBMI-for-age among overweight children post-pandemic. Our observation aligns with that of Weaver and colleagues, who found a significant acceleration in zBMI values in children with normal weight but not in overweight or obese children (43). Discrepancies between norm weight and overweight children have been corroborated by a meta-analysis (44). Arguably, these differences might stem from pre-pandemic behavioral patterns, with overweight children engaging in less health-supporting lifestyles that in turn, were not impacted by the pandemic. Because our study is intertwined with the COVID-19 pandemic, the extent to which the observed changes follow a secular trend or result from exposure to the epidemic remains unclear. Thus, future research should look into the long-term consequences of the COVID-19 pandemic both in healthy and vulnerable children.

Finally, we aimed to evaluate whether the effects observed in at-risk children post-intervention could be maintained over time. In line with previous research, we could not detect any outcome differences between the intervention and control groups at the 2 year follow-up (45). However, the scarcity of evidence on long-term intervention effectiveness makes it difficult to compare our results to others. As pointed out by one systematic review “more research is required with long-term follow-up to study the sustainability of (initial positive) changes” (46). Furthermore, the COVID-19 pandemic forced the closure of South African schools between March and June 2020. Upon reopening, social distancing protocols were put in place and the academic curriculum was trimmed to make up for the loss in schooling days (47). Consequently, it is likely that physical education has been neglected during this period. Thus, we cannot conclude whether the lack of long-term effect lies on the intervention itself, or rather its forced discontinuation due to the COVID-19 pandemic and the consequences thereof. These findings support the notion that systematic changes that integrate effective interventions into the structure of organizations are needed to ensure maintenance of positive effects. This is especially true in challenging settings like ours, where possibilities for PA are scarce.

### 4.1. Strengths and limitations

Strengths of this study include the use of a RCT with a longitudinal design, that allowed us to follow-up a vulnerable population including pre- and post-COVID-19 measurements. Furthermore, the availability of quality data, especially device-based PA, which is rare in relatively large sample sizes and low-income settings like ours. However, our study also had some limitations. First, intervention schools participated in different conditions with three of four schools receiving external support, while intervention fidelity was not assessed. Nevertheless, the application of mixed linear models enabled us to account for the variability between schools, classes, and intervention delivery. Second, low accelerometer wear-times might lead to inaccurate results. We approach this limitation by only including valid and representative days and controlling for wear-time in the analyses. Third, choosing appropriate cardiovascular risk markers and defining accurate cut-off points in children is a topic of debate in the literature. Different markers exist for different outcomes, and some change with age and maturation state of the child. Thus, using the same cut-off point for all children might present an under- or overestimation of the actual risk. This is partially overcome by the use of age- and sex-adjusted percentiles for BMI and BP, while none are available for glucose or lipids. Finally, as our study focuses on health outcomes of especially vulnerable children, no long-term data from healthy children was available to compare the development of cardiometabolic markers over time.

## 5. Conclusion

Our intervention was especially effective in improving high-intensity PA during school hours among less active children. It also improved cardiovascular risk factors, while benefit was higher for healthy children compared to at-risk children. Because we saw a deterioration in the long-term health outcomes of high-risk children, future studies should identify interventions that target the specific needs of vulnerable subgroups. Furthermore, intervention effects were lost in the long-term, possibly due to the program discontinuation during the COVID-19 pandemic. Moving forward, policymaking should provide for the integration of evidence-based interventions into resilient frameworks. This is of paramount importance if we aim to translate short-term benefits into long-lasting impact, especially for vulnerable populations living in challenging environments. In conclusion, schools are key settings to promote PA and improve health, but structural changes are necessary to ensure that effective interventions reach marginalized school populations and achieve sustainable impact.

## Data availability statement

The raw data supporting the conclusions of this article will be made available by the authors, without undue reservation.

## Ethics statement

The studies involving human participants were reviewed and approved by Nelson Mandela University Human Ethics Committee (ref. no. H18-HEA-HMS-001 and H20-HEA-HMS-001), Eastern Cape Department of Education, Eastern Cape Department of Health Ethics Committee (EC_201804_00), and Northwest and Central Switzerland (ref. no. R-2018-00047 and Req-2020-00430). Written informed consent to participate in this study was provided by the participants’ legal guardian/next of kin, while oral assent was sought from children.

## Author contributions

PA, IM, RR, CW, UP, MG, HS, PS, JU, LA, JD, DD, NJ, MN, and SN designed the research and intervention. PA, IM, LA, JD, DD, NJ, MN, and SN conducted the research. PA conceptualized the study, analyzed and interpreted the data, and wrote the manuscript. HS supported with data analysis. All authors contributed to the article and approved the submitted version.

## Funding

This research was financially supported by the Novartis Foundation (Basel, Switzerland) and the Swiss National Science Foundation (Bern, Switzerland; grant no. 192651), and took place under the auspices of the UNESCO Chair on “Physical Activity and Health in Educational Settings” (https://unesco-chair.dsbg.unibas.ch/en/). The funder had no role in study design, data collection, data analysis or data interpretation, nor the decision to submit the paper for publication.

## Conflict of interest

The authors declare that the research was conducted in the absence of any commercial or financial relationships that could be construed as a potential conflict of interest.

## Publisher’s note

All claims expressed in this article are solely those of the authors and do not necessarily represent those of their affiliated organizations, or those of the publisher, the editors and the reviewers. Any product that may be evaluated in this article, or claim that may be made by its manufacturer, is not guaranteed or endorsed by the publisher.

## Footnotes

^1^https://www.kazibantu.org/

## References

[1] Stanaway JD Afshin A Gakidou E Lim SS Abate D Abate KH. Global, regional, and national comparative risk assessment of 84 Behavioural, environmental and occupational, and metabolic risks or clusters of risks for 195 countries and territories, 1990–2017: a systematic analysis for the global burden of disease study 2017. Lancet. (2018) 392:1923–94. doi: 10.1016/s0140-6736(18)32225-6, PMID: 30496105 PMC6227755

[2] World Health Organization. Health for the world’s adolescents: a second chance in the second decade WHO (2014). Available at: https://www.who.int/publications/i/item/WHO-FWC-MCA-14.05

[3] Guthold R Stevens GA Riley LM Bull FC. Global trends in insufficient physical activity among adolescents: a pooled analysis of 298 population-based surveys with 1·6 million participants. Lancet Child Adolesc Health. (2020) 4:23–35. doi: 10.1016/s2352-4642(19)30323-2, PMID: 31761562 PMC6919336

[4] World Health Organization (2021). Obesity and overweight updated. WHO. (Accessed on June 9, 2021). Available at: https://www.who.int/news-room/fact-sheets/detail/obesity-and-overweight

[5] Song P Zhang Y Yu J Zha M Zhu Y Rahimi K. Global prevalence of hypertension in children: a systematic review and meta-analysis. JAMA Pediatr. (2019) 173:1154–63. doi: 10.1001/jamapediatrics.2019.3310, PMID: 31589252 PMC6784751

[6] Watkins D Hale J Hutchinson B Kataria I Kontis V Nugent R. Investing in non-communicable disease risk factor control among adolescents worldwide: a modelling study. BMJ Glob Health. (2019) 4:e001335. doi: 10.1136/bmjgh-2018-001335, PMID: 31139451 PMC6509594

[7] World Health Organization. (2022). Physical activity. WHO. (Accessed on October 5, 2022). Available at: https://www.who.int/news-room/fact-sheets/detail/physical-activity

[8] Janssen I LeBlanc AG. Systematic review of the health benefits of physical activity and fitness in school-aged children and youth. Int J Behav Nutr Phys Act. (2010) 7:40. doi: 10.1186/1479-5868-7-40, PMID: 20459784 PMC2885312

[9] Salvini M Gall S Müller I Walter C du Randt R Steinmann P. Physical activity and health-related quality of life among schoolchildren from disadvantaged Neighbourhoods in Port Elizabeth. South Africa Qual Life Res. (2018) 27:205–16. doi: 10.1007/s11136-017-1707-1, PMID: 28965191

[10] Gerber M Müller I Walter C du Randt R Adams L Gall S. Physical activity and dual disease burden among South African primary schoolchildren from disadvantaged neighbourhoods. Prev Med. (2018) 112:104–10. doi: 10.1016/j.ypmed.2018.04.001, PMID: 29626554

[11] World Health Organization. Saving lives, spending less new WHO investment case for NCDs WHO (2020). Available at: https://www.who.int/publications/i/item/WHO-NMH-NVI-18.8

[12] Carson RL Castelli DM Beighle A Erwin H. School-based physical activity promotion: a conceptual framework for research and practice. Child Obes. (2014) 10:100–6. doi: 10.1089/chi.2013.0134, PMID: 24655311

[13] Jones M Defever E Letsinger A Steele J Mackintosh KA. A mixed-studies systematic review and meta-analysis of school-based interventions to promote physical activity and/or reduce sedentary time in children. J Sport Health Sci. (2020) 9:3–17. doi: 10.1016/j.jshs.2019.06.009, PMID: 31921476 PMC6943767

[14] Love R Adams J van Sluijs EMF. Are school-based physical activity interventions effective and equitable? A meta-analysis of cluster randomized controlled trials with accelerometer-assessed activity. Obes Rev. (2019) 20:859–70. doi: 10.1111/obr.12823, PMID: 30628172 PMC6563481

[15] Pfledderer CD Burns RD Byun W Carson RL Welk GJ Brusseau TA. School-based physical activity interventions in rural and urban/suburban communities: a systematic review and meta-analysis. Obes Rev. (2021) 22:e13265. doi: 10.1111/obr.13265 33938109

[16] Errisuriz VL Golaszewski NM Born K Bartholomew JB. Systematic review of physical education-based physical activity interventions among elementary school children. J Prim Prev. (2018) 39:303–27. doi: 10.1007/s10935-018-0507-x, PMID: 29705883

[17] Nguyen S Hacker AL Henderson M Barnett T Mathieu ME Pagani L. Physical activity programs with post-intervention follow-up in children: a comprehensive review according to categories of intervention. Int J Environ Res Public Health. (2016) 13:664. doi: 10.3390/ijerph13070664, PMID: 27376315 PMC4962205

[18] Müller I Schindler C Adams L Endes K Gall S Gerber M. Effect of a multidimensional physical activity intervention on body mass index, skinfolds and fitness in South African children: results from a cluster-randomised controlled trial. Int J Environ Res Public Health. (2019) 16:232. doi: 10.3390/ijerph16020232, PMID: 30650624 PMC6352127

[19] Uys M Draper CE Hendricks S de Villiers A Fourie J Steyn NP. Impact of a South African school-based intervention, healthkick, on fitness correlates. Am J Health Behav. (2016) 40:55–66. doi: 10.5993/AJHB.40.1.7, PMID: 26685814

[20] Adom T De Villiers A Puoane T Kengne AP. School-based interventions targeting nutrition and physical activity, and body weight status of African children: a systematic review. Nutrients. (2019) 12:95. doi: 10.3390/nu12010095, PMID: 31905832 PMC7019429

[21] Lambrinou CP Androutsos O Karaglani E Cardon G Huys N Wikstrom K. Effective strategies for childhood obesity prevention via school based, family involved interventions: a critical review for the development of the Feel4Diabetes-study school based component. BMC Endocr Disord. (2020) 20:52. doi: 10.1186/s12902-020-0526-5, PMID: 32370795 PMC7201517

[22] Müller I Smith D Adams L Aerts A Damons BP Degen J. Effects of a school-based health intervention program in marginalized communities of Port Elizabeth, South Africa (the Kazibantu study): protocol for a randomized controlled trial. JMIR Res Protoc. (2019) 8:e14097. doi: 10.2196/14097, PMID: 31298224 PMC6657454

[23] Arnaiz P Adams L Müller I Gerber M Walter C du Randt R. Sustainability of a school-based health intervention for prevention of non-communicable diseases in marginalised communities: protocol for a mixed-methods cohort study. BMJ Open. (2021) 11:e047296. doi: 10.1136/bmjopen-2020-047296, PMID: 34610931 PMC8493924

[24] Troiano RP Berrigan D Dodd KW Masse LC Tilert T McDowell M. Physical activity in the United States measured by accelerometer. Med Sci Sports Exerc. (2008) 40:181–8. doi: 10.1249/mss.0b013e31815a51b3 18091006

[25] World Health Organization. Growth reference data for 5–19 years: BMI-for-age: World Health Organization (2007), (2020). (Accessed on September 13, 2020). Available at: https://www.who.int/growthref/who2007_bmi_for_age/en/

[26] Villa JK Silva AR Santos TS Ribeiro AQ Sant'Ana LF. Metabolic syndrome risk assessment in children: use of a single score. Rev Paul Pediatr. (2015) 33:187–93. doi: 10.1016/j.rpped.2014.11.001, PMID: 25649382 PMC4516373

[27] Flynn JT Kaelber DC Baker-Smith CM Blowey D Carroll AE Daniels SR. Clinical practice guideline for screening and management of high blood pressure in children and adolescents. Pediatrics. (2017) 140:e20171904. doi: 10.1542/peds.2017-1904 28827377

[28] Association AD. 2. Classification and diagnosis of diabetes: standards of medical care in diabetes-2020. Diabetes Care. (2020) 43:S14–31. doi: 10.2337/dc20-S002, PMID: 31862745

[29] Expert Panel on Integrated Guidelines for Cardiovascular Health and Risk Reduction in Children and Adolescents and National Heart, Lung, and Blood Institute. Expert panel on integrated guidelines for cardiovascular health and risk reduction in children and adolescents: summary report. Pediatrics. (2011) 128:S213–56. doi: 10.1542/peds.2009-2107C, PMID: 22084329 PMC4536582

[30] Gall S Adams L Joubert N Ludyga S Müller I Nqweniso S. Effect of a 20-week physical activity intervention on selective attention and academic performance in children living in disadvantaged neighborhoods: a cluster randomized control trial. PLoS One. (2018) 13:e0206908. doi: 10.1371/journal.pone.0206908, PMID: 30408073 PMC6224098

[31] Cohen J. Statistical power analysis for the behavioral sciences. 2nd ed Lawrence Erlbaum (1988).

[32] Mannocci A D’Egidio V Backhaus I Federici A Sinopoli A Ramirez Varela A. Are there effective interventions to increase physical activity in children and young people? An umbrella review. Int J Environ Res Public Health. (2020) 17:3528. doi: 10.3390/ijerph17103528, PMID: 32443505 PMC7277151

[33] Hartwig TB Sanders T Vasconcellos D Noetel M Parker PD Lubans DR. School-based interventions modestly increase physical activity and cardiorespiratory fitness but are least effective for youth who need them most: an individual participant pooled analysis of 20 controlled trials. Br J Sports Med. (2021) 55:721–9. doi: 10.1136/bjsports-2020-102740, PMID: 33441332

[34] Bailey R Wellard I Dismore H. Girls’ participation in physical activities and sports: benefits, patterns, influences and ways forward In: . Technical Report: World Health Organisation (2004)

[35] Tarp J Child A White T Westgate K Bugge A Grontved A. Physical activity intensity, bout-duration, and cardiometabolic risk markers in children and adolescents. Int J Obes. (2018) 42:1639–50. doi: 10.1038/s41366-018-0152-8, PMID: 30006582 PMC6160399

[36] de Ferranti SD Steinberger J Ameduri R Baker A Gooding H Kelly AS. Cardiovascular risk reduction in high-risk pediatric patients: a scientific statement from the American Heart Association. Circulation. (2019) 139:e603–34. doi: 10.1161/CIR.0000000000000618, PMID: 30798614

[37] Salway R Foster C de Vocht F Tibbitts B Emm-Collison L House D. Accelerometer-measured physical activity and sedentary time among children and their parents in the UK before and after COVID-19 lockdowns: a natural experiment. Int J Behav Nutr Phys Act. (2022) 19:51. doi: 10.1186/s12966-022-01290-4, PMID: 35570265 PMC9107948

[38] Schefelker J Zhang X Dodge A Marten K Dimailig G Bartlett HL. Impact of the COVID-19 pandemic on cardiometabolic health parameters in children with preexisting dyslipidemia. J Clin Lipidol. (2022) 16:643–8. doi: 10.1016/j.jacl.2022.06.006, PMID: 35798651 PMC9232258

[39] Shah N Khadilkar V Oza C Karguppikar M Bhor S Ladkat D. Impact of decreased physical activity due to COVID restrictions on cardio-metabolic risk parameters in Indian children and youth with type 1 diabetes. Diabetes Metab Syndr. (2022) 16:102564. doi: 10.1016/j.dsx.2022.102564, PMID: 35816949

[40] Marks BE Khilnani A Meyers A Estrada DE Boughton J Gai J. 15-OR: the COVID-19 pandemic and new-onset pediatric type 2 diabetes. Diabetes. (2021):70. doi: 10.2337/db21-15-OR

[41] Karatzi K Poulia KA Papakonstantinou E Zampelas A. The impact of nutritional and lifestyle changes on body weight, body composition and cardiometabolic risk factors in children and adolescents during the pandemic of COVID-19: a systematic review. Children. (2021) 8:1130. doi: 10.3390/children8121130, PMID: 34943326 PMC8700559

[42] Owen CG Nightingale CM Rudnicka AR Sattar N Cook DG Ekelund U. Physical activity, obesity and cardiometabolic risk factors in 9- to 10-year-old UK children of White European, South Asian and Black African-Caribbean origin: the child heart and health study in England (chase). Diabetologia. (2010) 53:1620–30. doi: 10.1007/s00125-010-1781-1, PMID: 20454952 PMC2892063

[43] Weaver RG Hunt ET Armstrong B Beets MW Brazendale K Turner-McGrievy G. COVID-19 leads to accelerated increases in Children’s BMI *Z*-score gain: an interrupted time-series study. Am J Prev Med. (2021) 61:e161–9. doi: 10.1016/j.amepre.2021.04.007, PMID: 34148734 PMC8443301

[44] Chang TH Chen YC Chen WY Chen CY Hsu WY Chou Y. Weight gain associated with COVID-19 lockdown in children and adolescents: a systematic review and meta-analysis. Nutrients. (2021) 13:3668. doi: 10.3390/nu13103668, PMID: 34684669 PMC8540321

[45] McEwan D Rhodes RE Beauchamp MR. What happens when the party is over?: sustaining physical activity behaviors after intervention cessation. Behav Med. (2020) 48:1–9. doi: 10.1080/08964289.2020.1750335, PMID: 32275199

[46] Singh A Bassi S Nazar GP Saluja K Park M Kinra S. Impact of school policies on non-communicable disease risk factors—a systematic review. BMC Public Health. (2017) 17:292. doi: 10.1186/s12889-017-4201-3, PMID: 28376833 PMC5379668

[47] Ramrathan L. School curriculum in South Africa in the COVID-19 context: an opportunity for education for relevance. Prospects. (2021) 51:383–92. doi: 10.1007/s11125-020-09490-1, PMID: 32836431 PMC7406695

