# Supplementary material for: Intervention effects and long-term changes in physical activity and cardiometabolic outcomes among children at risk of noncommunicable diseases in South Africa: a cluster-randomized controlled trial and follow-up analysis
Source: Front Public Health. 2023 May 26;11:1199381. doi: 10.3389/fpubh.2023.1199381 (PMC10250595; doi:10.3389/fpubh.2023.1199381)
Supplement: Supplementary file 1 [file Table_1.DOCX]

Supplementary Material

# Supplementary Tables

| **Table S1** **\|** Post-hoc mixed linear regression analysis for intervention effect on physical activity between physically active and inactive children for girls and boys separately. | | | | | | | | |
| --- | --- | --- | --- | --- | --- | --- | --- | --- |
| ***Risk factor*** | ***Physically active*** | | | | ***Physically inactive*** | | | |
|  | ***N*** | ***Beta*** | ***95% CI*** | ***p-value*** | ***N*** | ***Beta*** | ***95% CI*** | ***p-value*** |
| Total MVPA^a^ (min/day) |  |  |  |  |  |  |  |  |
| Girls | 163 | -0.60 | -6.06 – 5.21 | 0.785 | 189 | 1.42 | -2.85 – 6.27 | 0.348 |
| Boys | 262 | -3.76 | -9.67 – 2.02 | 0.078 | 65 | -.89 | -12.06 – 10.28 | 0.851 |
| School MVPA^a^ (min/day) |  |  |  |  |  |  |  |  |
| Girls | 195 | **1.80** | **-0.22 – 3.82** | **0.035** | 214 | **2.03** | **0.58 – 3.42** | **<0.001** |
| Boys | 314 | -1.39 | -3.29 – 0.50 | 0.109 | 77 | 0.68 | -5.39 – 7.82 | 0.694 |

*Note.* Physical inactivity defined as below 60 min of MVPA a day. Controlled for baseline outcome, wear-time, age, sex, and SES using class as random effects. Bias-corrected and accelerated (BCa) with 1,000 replicates bootstrap adjusted results.

^a^Moderate-to-vigorous physical activity.

| **Table S2 \|** Linear mixed models for intervention effect on cardiometabolic risk factors in children at-risk of noncommunicable disorders from post-intervention to follow-up. | | | | |
| --- | --- | --- | --- | --- |
| ***Risk factor*** | ***N*** | ***Beta*** | ***95% CI*** | ***p-value*** |
| Total MVPA^a^ (min/day) | 59 | 3.33 | -8.93 – 17.26 | 0.416 |
| School MVPA (min/day) | 72 | -1.27 | -5.25 – 2.67 | 0.341 |
| BMI-for-age^b^ (z scores) | 93 | -0.13 | -0.19 – 0.22 | 0.885 |
| MAP^c^ (mmHg) | 139 | 0.80 | -1.54 – 3.38 | 0.439 |
| HbA1c^d^ (mmol/mol) | 40 | 0.85 | -0.47 – 2.32 | 0.121 |
| TC:HDL^e^ | 55 | -0.03 | -0.31 – 0.28 | 0.830 |

*Note.* At-risk is defined for each outcome separately as: physical inactivity for total and school MVPA, overweight or obesity for BMI-for-age, pre- or hypertension for MAP, pre- or diabetes for HbA1c, pre- or dyslipidemia for TC:HD. All outcomes have been controlled for baseline outcome, age, sex and SES using class as random effects; BMI-for-age has not been controlled for age and sex; total and school MVPA have been further controlled for wear-time. Bias-corrected and accelerated (BCa) with 1,000 replicates bootstrap adjusted results.

^a^Moderate-to-vigorous physical activity. ^b^Body mass index. ^c^Mean arterial pressure. ^d^Glycated haemoglobin. ^e^Total cholesterol to high-density lipoprotein ratio.

| Table S3 \| CONSORT 2010 checklist of information to include when reporting a randomised trial. | | | |
| --- | --- | --- | --- |
| Section/Topic | Item No | Checklist item | Page No |
| Title and abstract | | | |
|  | 1a | Identification as a randomised trial in the title | 1 |
|  | 1b | Structured summary of trial design, methods, results, and conclusions (for specific guidance see CONSORT for abstracts) | 1 |
| Introduction | | | |
| Background and objectives | 2a | Scientific background and explanation of rationale | 2 |
|  | 2b | Specific objectives or hypotheses | 2 |
| Methods | | | |
| Trial design | 3a | Description of trial design (such as parallel, factorial) including allocation ratio | 2,3 |
|  | 3b | Important changes to methods after trial commencement (such as eligibility criteria), with reasons | n/a |
| Participants | 4a | Eligibility criteria for participants | 3 |
|  | 4b | Settings and locations where the data were collected | 3 |
| Interventions | 5 | The interventions for each group with sufficient details to allow replication, including how and when they were actually administered | 3 |
| Outcomes | 6a | Completely defined pre-specified primary and secondary outcome measures, including how and when they were assessed | 3 |
|  | 6b | Any changes to trial outcomes after the trial commenced, with reasons | n/a |
| Sample size | 7a | How sample size was determined | 3 |
|  | 7b | When applicable, explanation of any interim analyses and stopping guidelines | n/a |
| Randomisation: |  |  |  |
| Sequence generation | 8a | Method used to generate the random allocation sequence | 3 |
|  | 8b | Type of randomisation; details of any restriction (such as blocking and block size) | 3 |
| Allocation concealment mechanism | 9 | Mechanism used to implement the random allocation sequence (such as sequentially numbered containers), describing any steps taken to conceal the sequence until interventions were assigned | 3 |
| Implementation | 10 | Who generated the random allocation sequence, who enrolled participants, and who assigned participants to interventions | 3 |
| Blinding | 11a | If done, who was blinded after assignment to interventions (for example, participants, care providers, those assessing outcomes) and how | n/a |
|  | 11b | If relevant, description of the similarity of interventions | n/a |
| Statistical methods | 12a | Statistical methods used to compare groups for primary and secondary outcomes | 4,5 |
|  | 12b | Methods for additional analyses, such as subgroup analyses and adjusted analyses | 4,5 |
| Results | | | |
| Participant flow (a diagram is strongly recommended) | 13a | For each group, the numbers of participants who were randomly assigned, received intended treatment, and were analysed for the primary outcome | 3 |
|  | 13b | For each group, losses and exclusions after randomisation, together with reasons | 3 |
| Recruitment | 14a | Dates defining the periods of recruitment and follow-up | 3 |
|  | 14b | Why the trial ended or was stopped | 3 |
| Baseline data | 15 | A table showing baseline demographic and clinical characteristics for each group | 5 |
| Numbers analysed | 16 | For each group, number of participants (denominator) included in each analysis and whether the analysis was by original assigned groups | 5,6 |
| Outcomes and estimation | 17a | For each primary and secondary outcome, results for each group, and the estimated effect size and its precision (such as 95% confidence interval) | 5,6 |
|  | 17b | For binary outcomes, presentation of both absolute and relative effect sizes is recommended | n/a |
| Ancillary analyses | 18 | Results of any other analyses performed, including subgroup analyses and adjusted analyses, distinguishing pre-specified from exploratory | 6 |
| Harms | 19 | All important harms or unintended effects in each group (for specific guidance see CONSORT for harms) | n/a |
| Discussion | | | |
| Limitations | 20 | Trial limitations, addressing sources of potential bias, imprecision, and, if relevant, multiplicity of analyses | 8 |
| Generalisability | 21 | Generalisability (external validity, applicability) of the trial findings | 8 |
| Interpretation | 22 | Interpretation consistent with results, balancing benefits and harms, and considering other relevant evidence | 8,9 |
| Other information | | |  |
| Registration | 23 | Registration number and name of trial registry | 9 |
| Protocol | 24 | Where the full trial protocol can be accessed, if available | 3 |
| Funding | 25 | Sources of funding and other support (such as supply of drugs), role of funders | 9 |

| Table S4 \| STROBE Statement—Checklist of items that should be included in reports of cohort studies. | | | |
| --- | --- | --- | --- |
|  | Item No | Recommendation | Page No |
| **Title and abstract** | 1 | (*a*) Indicate the study’s design with a commonly used term in the title or the abstract | 1 |
|  |  | (*b*) Provide in the abstract an informative and balanced summary of what was done and what was found | 1 |
| Introduction | | | |
| Background/rationale | 2 | Explain the scientific background and rationale for the investigation being reported | 2 |
| Objectives | 3 | State specific objectives, including any prespecified hypotheses | 2 |
| Methods | | | |
| Study design | 4 | Present key elements of study design early in the paper | 3 |
| Setting | 5 | Describe the setting, locations, and relevant dates, including periods of recruitment, exposure, follow-up, and data collection | 3 |
| Participants | 6 | (*a*) Give the eligibility criteria, and the sources and methods of selection of participants. Describe methods of follow-up | 3 |
|  |  | (*b*) For matched studies, give matching criteria and number of exposed and unexposed | n/a |
| Variables | 7 | Clearly define all outcomes, exposures, predictors, potential confounders, and effect modifiers. Give diagnostic criteria, if applicable | 4 |
| Data sources/ measurement | 8* | For each variable of interest, give sources of data and details of methods of assessment (measurement). Describe comparability of assessment methods if there is more than one group | 4 |
| Bias | 9 | Describe any efforts to address potential sources of bias | 3,4 |
| Study size | 10 | Explain how the study size was arrived at | 3 |
| Quantitative variables | 11 | Explain how quantitative variables were handled in the analyses. If applicable, describe which groupings were chosen and why | 4,5 |
| Statistical methods | 12 | (*a*) Describe all statistical methods, including those used to control for confounding | 4,5 |
|  |  | (*b*) Describe any methods used to examine subgroups and interactions | 4,5 |
|  |  | (*c*) Explain how missing data were addressed | 4,5 |
|  |  | (*d*) If applicable, explain how loss to follow-up was addressed | 4,5 |
|  |  | (*e*) Describe any sensitivity analyses | n/a |
| Results | | |  |
| Participants | 13* | (a) Report numbers of individuals at each stage of study—eg numbers potentially eligible, examined for eligibility, confirmed eligible, included in the study, completing follow-up, and analysed | 3 |
|  |  | (b) Give reasons for non-participation at each stage | 3 |
|  |  | (c) Consider use of a flow diagram | 3 |
| Descriptive data | 14* | (a) Give characteristics of study participants (eg demographic, clinical, social) and information on exposures and potential confounders | 5 |
|  |  | (b) Indicate number of participants with missing data for each variable of interest | 5 |
|  |  | (c) Summarise follow-up time (eg, average and total amount) | 5 |
| Outcome data | 15* | Report numbers of outcome events or summary measures over time | n/a |
| Main results | 16 | (*a*) Give unadjusted estimates and, if applicable, confounder-adjusted estimates and their precision (eg, 95% confidence interval). Make clear which confounders were adjusted for and why they were included | 5,6 |
|  |  | (*b*) Report category boundaries when continuous variables were categorized | n/a |
|  |  | (*c*) If relevant, consider translating estimates of relative risk into absolute risk for a meaningful time period | n/a |
| Other analyses | 17 | Report other analyses done—eg analyses of subgroups and interactions, and sensitivity analyses | 6 |
| Discussion | | | |
| Key results | 18 | Summarise key results with reference to study objectives | 6 |
| Limitations | 19 | Discuss limitations of the study, taking into account sources of potential bias or imprecision. Discuss both direction and magnitude of any potential bias | 8 |
| Interpretation | 20 | Give a cautious overall interpretation of results considering objectives, limitations, multiplicity of analyses, results from similar studies, and other relevant evidence | 8,9 |
| Generalisability | 21 | Discuss the generalisability (external validity) of the study results | 8,9 |
| Other information | | | |
| Funding | 22 | Give the source of funding and the role of the funders for the present study and, if applicable, for the original study on which the present article is based | 9 |
